# Unsupervised machine learning effectively clusters pediatric spastic cerebral palsy patients for determination of optimal responders to selective dorsal rhizotomy

**DOI:** 10.1038/s41598-023-35021-x

**Published:** 2023-05-19

**Authors:** Xiaobin Hou, Yanyun Yan, Qijia Zhan, Junlu Wang, Bo Xiao, Wenbin Jiang

**Affiliations:** 1 Department of Neurosurgery, Shanghai Children’s Hospital, School of Medicine, Shanghai Jiao Tong University, 355 Luding Road, Shanghai, 200062 China; 2 Department of Operating Room, Shanghai Children’s Hospital, School of Medicine, Shanghai Jiao Tong University, Shanghai, China

**Keywords:** Paediatric research, Movement disorders

## Abstract

Selective dorsal rhizotomy (SDR) can reduce the spasticity in patients with spastic cerebral palsy (SCP) and thus improve the motor function in these patients, but different levels of improvement in motor function were observed among patients after SDR. The aim of the present study was to subgroup patients and to predict the possible outcome of SDR based on the pre-operational parameters. A hundred and thirty-five pediatric patients diagnosed with SCP who underwent SDR from January 2015 to January 2021 were retrospectively reviewed. Spasticity of lower limbs, the number of target muscles, motor functions, and other clinical parameters were used as input variables for unsupervised machine learning to cluster all included patients. The postoperative motor function change is used to assess the clinical significance of clustering. After the SDR procedure, the spasticity of muscles in all patients was reduced significantly, and the motor function was promoted significantly at the follow-up duration. All patients were categorized into three subgroups by both hierarchical and K-means clustering methods. The three subgroups showed significantly different clinical characteristics except for the age at surgery, and the post-operational motor function change at the last follow-up in these three clusters was different. Three subgroups clustered by two methods could be identified as “best responders”, “good responders” and “moderate responders” based on the increasement of motor function after SDR. Clustering results achieved by hierarchical and K-means algorithms showed high consistency in subgrouping the whole group of patients. These results indicated that SDR could relieve the spasticity and promote the motor function of patients with SCP. Unsupervised machine learning methods can effectively and accurately cluster patients into different subgroups suffering from SCP based on pre-operative characteristics. Machine learning can be used for the determination of optimal responders for SDR surgery.

## Introduction

Treatment approaches for children with spastic cerebral palsy (SCP) are aimed to promote the moving abilities of them^1^. Several studies reported that selective dorsal rhizotomy (SDR) is a safe procedure for reducing spasticity of lower extremities in pediatric SCP^2–4^. The motor function of these children is significantly improved after SDR when supplemented with rehabilitation therapy^5^. Patients who is about to accept SDR procedure should meet the surgical indications, which has been established for decades^6^. Nonetheless, it is reported that different levels of increased motor function were observed among patients after undergoing SDR procedure^7^. Predicting the possible prognosis of SDR basing on certain pre-operational parameters is of great clinical importance.

Machine learning is used for analysis of real-life data using mathematical models^8^. These mathematical methods can be used for analysis of data in different fields. Machine learning methods have been used in clinical practice for diagnosis, classification and evaluation of heart failure patients with good results^9^. Therefore, machine learning can also be used for classification of SCP patients before undergoing SDR surgery. Machine learning comprises supervised or unsupervised method, depending on the algorithm used^10^. Unsupervised machine learning is used for sorting the entire dataset into several subsets based on the level of similarity^11^. The data in this case has no target attributes, and the computer should determine the inherent structure and patterns of the dataset. The differences between several sorted subsets are clarified by evaluating the characteristics of the different subsets.

The present study sought to classify children suffering from SCP using the unsupervised machine learning method to identify subjects eligible for SDR surgery and improve the outcomes of patients who undergo the procedure.

## Materials and methods

### Research population, SDR procedure, post-operational rehabilitation program and follow up

A retrospective study was conducted in consecutive pediatric patients diagnosed with SCP who treated in our department from January 2015 to January 2021. Diagnosis of SCP was conducted by multi-disciplinary treatment experts. Patients who were met the SDR indications received single-level approach SDR at the lumbar segment. The relevant techniques of SDR surgery have been elaborated in detail in our previously published articles^2,7^. In brief, the child was placed in a prone position with the head lowered, and the surgical incision was typically made at the L2–L3 interspace. After laminectomy, we made an incision of about 1.2 cm in the midline of the dura mater. With guidance from neurophysiological monitoring, we carefully test every nerve root/rootlet in the surgical area and to transect or protect nerve fibers according to the rhizotomy protocol^7^. The post-operative rehabilitation program was applied to these children 3 days after SDR (Supplementary Fig. 1). In detail, strengthening program starts 3 days after the operation, and the balancing program starts 7 days after SDR. Ambulating program starts 3 months, 6 months, 12 months, 18 months after SDR in children classified as GMFCS I, II, III and IV, respectively. Children after SDR were suggested to have follow-up every 3–6 months for the assessment of spasticity and motor function, as well as adjusting rehabilitation program individually. Patients who had a follow-up duration longer than a year were included in this study.

### Assessment of spasticity

Muscle tone of muscles in bilateral lower extremities in all patients was pre-operatively and post-operatively assessed by one physiotherapist using the modified Ashworth Scale (MAS)^12^. The MAS score was used to determine the MAS grade, with a score 0 representing MAS grade 0 (no increase in muscle tone), a score of 1 indicating MAS grade 1 (slight increase in muscle tone, presented as a catch and release or by minimum resistance at the end of the range of motion when the affected part was moved in flexion or extension), a score of 2 represented a MAS grade 1^+^ (slight increase in muscle tone, exhibited as a catch, followed by minimal resistance throughout the remainder of the range of movement), a score of 3 represented MAS grade 2 (moderate increase in muscle tone), a score of 4 indicating a MAS grade 3 (significant increase in muscle tone) and a score of 5 denoting a MAS grade 4 (affected part rigid in flexion or extension)^13^. Muscles assessed in this study included bilateral hip adductors, hamstrings, gastrocnemius, and soleus. Muscles evaluated as MAS score 3 or higher before the SDR procedure were referred as target muscles.

### Evaluation of motor function

The motor function of all patients was examined by one single physiotherapist who conducted muscle tone assessment. The gross motor function classification system (GMFCS) and gross motor function measure-66 (GMFM-66) were utilized to assess the motion ability of the participants. GMFCS is a five-grade classification system for determination of motor function of patients presenting with level I (walk without limitations) to level V (dependent on humans and equipment) SCP^14,15^. GMFM-66 is an observational clinical tool for evaluation of motor function changes in cerebral palsy^16^. GMFM-66 scoring system is a four-point-scale comprising 66 items grouped into five dimensions of gross motor function. A 5-year-old child without motor disabilities exhibits the maximum score (a score of 100). GMFM-66 score is highly correlated with the GMFCS grade, but the score is more accurate as a tool for motor function evaluation compared with use of the classification system.

Considering that the motor function of children with spastic cerebral palsy would be improved owing to the natural growth, we utilized the equation to calculate the expected natural evolution score over a definite period of time, which is accessible online (http://www.gmfmer.ca)^17^. The expected natural evolution could serve as a historical control to measure and compare GMFM-66 score evolution in children with different GMFCS level, and thus normalizing the improvement achieved by SDR and the post-SDR rehabilitation program in the cohort. It has to be mentioned that the monthly expected natural evolution was calculated up to the age of 96 months, and the expected natural evolution of those older than 96 months were taken as zero in this study.

### Clinical characteristics, data processing, and unsupervised machine learning

Variables including age, pre-operational GMFCS level, GMFM-66 score, number of target muscles, and MAS scores of bilateral hip adductors, hamstrings, gastrocnemius, and soleus, were used as input data for unsupervised machine learning calculations. Correlation analyses were conducted between the input variables before machine learning analysis. GMFCS level was eliminated from the input data as the correlation coefficient between GMFCS score and GMFM-66 was more than 0.8 (absolute value). The input variables were scaled before clustering. Subsequently, hierarchical clustering and K-means clustering were performed for the eleven variables^18–21^. The elbow method was used to determine the “K” value of the variables before clustering. The elbow method comprises use of a metric for evaluation of reliability of the clustering outcome for various values of K and determining the elbow point. The elbow point refers to the iteration when there is no significant improvement in the clustering outcome. The clustering results were visualized by dimensionality reduction through principal component analysis^22^. Group sorting and principal component analysis were performed using “kmeans”, “hclust”, “psyc” and “clusterR” packages in R studio (version 4.1.3). After clustering, the post-operative GMFM-66 score change was taken as outcome measure to compare the effect of SDR among these three subgroups.

### Statistical analysis

Continuous variables with normal distribution were presented as mean ± SD, whereas variables that with skewed distribution were reported as median (Q1, Q3). For data that follow normal distribution, we used paired *t* test for statistical comparison, and for data that do not follow normal distribution, we employed Wilcoxon signed-rank test for comparison. For comparisons between subgroups, we use ANOVA with Tukey's test or Kruskal–Wallis test with Dunn's multiple comparison test as appropriate. A value of *p* < 0.05 was considered statistically significant. Data were analyzed by SPSS software version 24.0 for Windows (SPSS Inc., Chicago, IL, USA).

### Ethical approval

This study was conducted in accordance with the relevant guidelines and the Declaration of Helsinki. It is a retrospective study of clinical data and it has been approved by the Ethics Review Committee, Children’s Hospital of Shanghai, Shanghai Jiao Tong University (Approval No: 2020R069-E02). Because of the retrospective nature of the study, the informed consent for inclusion was waived by the ethics committee of Children’s Hospital of Shanghai.

## Results

### Demographic details of included patients

A total of 135 cases (99 boys and 36 girls) were included in the current study. The mean age of the participants was 6.0 ± 1.9 years old, with age of subjects at SDR ranging from 3 to 12 years. All cases were diagnosed with SCP, including 12 hemiplegias, 58 diplegias, and 65 quadriplegias, respectively. The median GMFCS level of the subjects before the operation was level 3, whereas the average GMFM-66 score was 55.8 ± 14.1. The pre-operative muscle tone of lower extremities of the patients was atypical. The median MAS score of bilateral adductors and hamstrings was 3.0, and the MAS score of distal muscles was markedly high with a score of 4.0 for bilateral gastrocnemius and bilateral soleus. Altogether, 6.5 ± 1.8 muscle groups were evaluated ≥ MAS score 3 during the physical assessment and were marked as target muscles.

### Surgical outcome of SDR

All the subjects underwent SDR. A mean of 66.4 ± 7.5 roots (rootlets) was tested during the surgery using the intraoperative neurophysiological monitoring system, and 11.1 ± 5.6 sensory rootlets were partially incised based on the rhizotomy protocol. The mean follow-up duration for the participants was 568 days, and the anticipated expected natural evolution according to the algorithm increased 1.03 in median value when compared with pre-operational status. During the post-operational assessment, the MAS score of muscles in lower extremities reduced significantly, which in detail, from 3 to 1 in right adductors, 3 to 1 in left adductors, 3 to 2 in right hamstrings, 3 to 2 in left hamstrings, 4 to 2 in right Gastrocnemii, 4 to 2 in left Gastrocnemii, 4 to 1 in right Soleus and 4 to 1 in left Soleus (Table 1). The GMFM-66 score increased by 6.3 in average at the last follow-up physical evaluation, and the median level of GMFCS increased from level 3 to level 2.

**Table 1:** Clinical parameters of included cases before and after selective dorsal rhizotomy.

Characteristics	Pre-op status	Post-op status	*p* value
Pre-op GMFM-66 score	55.8 ± 14.1	62.1 ± 15.7	< 0.0001
Pre-op GMFCS level	3.0 (2.0, 3.0)	2.0 (2.0, 3.0)	< 0.0001
Muscle tension (MAS score)			
AddR	3.0 (1.0, 4.0)	1.5 (0, 2.0)	< 0.0001
AddL	3.0 (1.0, 4.0)	1.0 (0, 2.0)	< 0.0001
HamR	3.0 (1.0, 3.0)	2.0 (0, 2.0)	< 0.0001
HamL	3.0 (1.0, 3.0)	2.0 (0, 2.0)	< 0.0001
GasR	4.0 (4.0, 5.0)	2.0 (2.0, 3.0)	< 0.0001
GasL	4.0 (3.0, 4.0)	2.0 (2.0, 3.0)	< 0.0001
SolR	4.0 (3.0, 4.0)	1.0 (1.0, 2.0)	< 0.0001
SolL	4.0 (3.0, 4.0)	1.0 (0, 1.0)	< 0.0001

*GMFM-66* gross motor function measure-66, *GMFCS* gross motor function classification system, *MAS* modified Ashworth Scale, *AddR* right adductor, *AddL* left adductor, *HamR* right hamstring, *HamL* left hamstring, *GasR* right gastrocnemius, *GasL* left gastrocnemius, *SolR* right soleus, *SolL* left soleus.

### Clustering and visualization of patients

**Figure 1:**
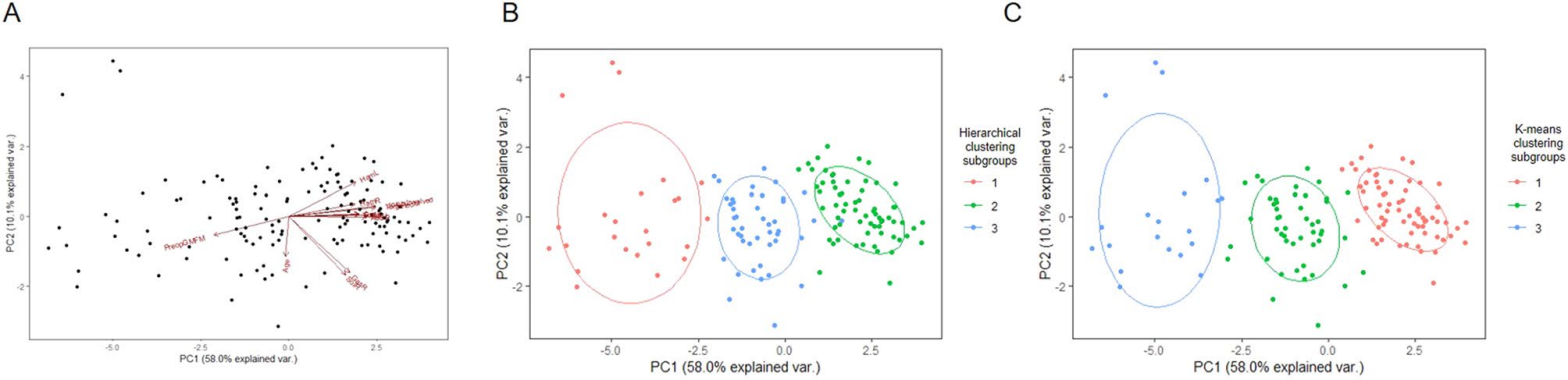
Visualization of realized by the principal component analysis (**A**) of the whole dataset and the clustering done by hierarchical clustering (**B**) and K-means clustering (**C**).

Eleven variables were used as input parameters for unsupervised machine learning (hierarchical clustering and K-means clustering). Principal component analysis was performed to reduce the data dimensionality to two principal components (PC1 and PC2) for visualization of the clustering results (Fig. 1A). The dendrogram generated by the hierarchical clustering (Supplementary Fig. 2A,B) and the result obtained by the elbow method (Supplementary Fig. 2C) both indicated that the whole dataset should be clustered into three subgroups. Grouping of the variables conducted through hierarchical clustering was consistent with the K-means clustering results (Fig. 1B,C).

**Figure 2:**
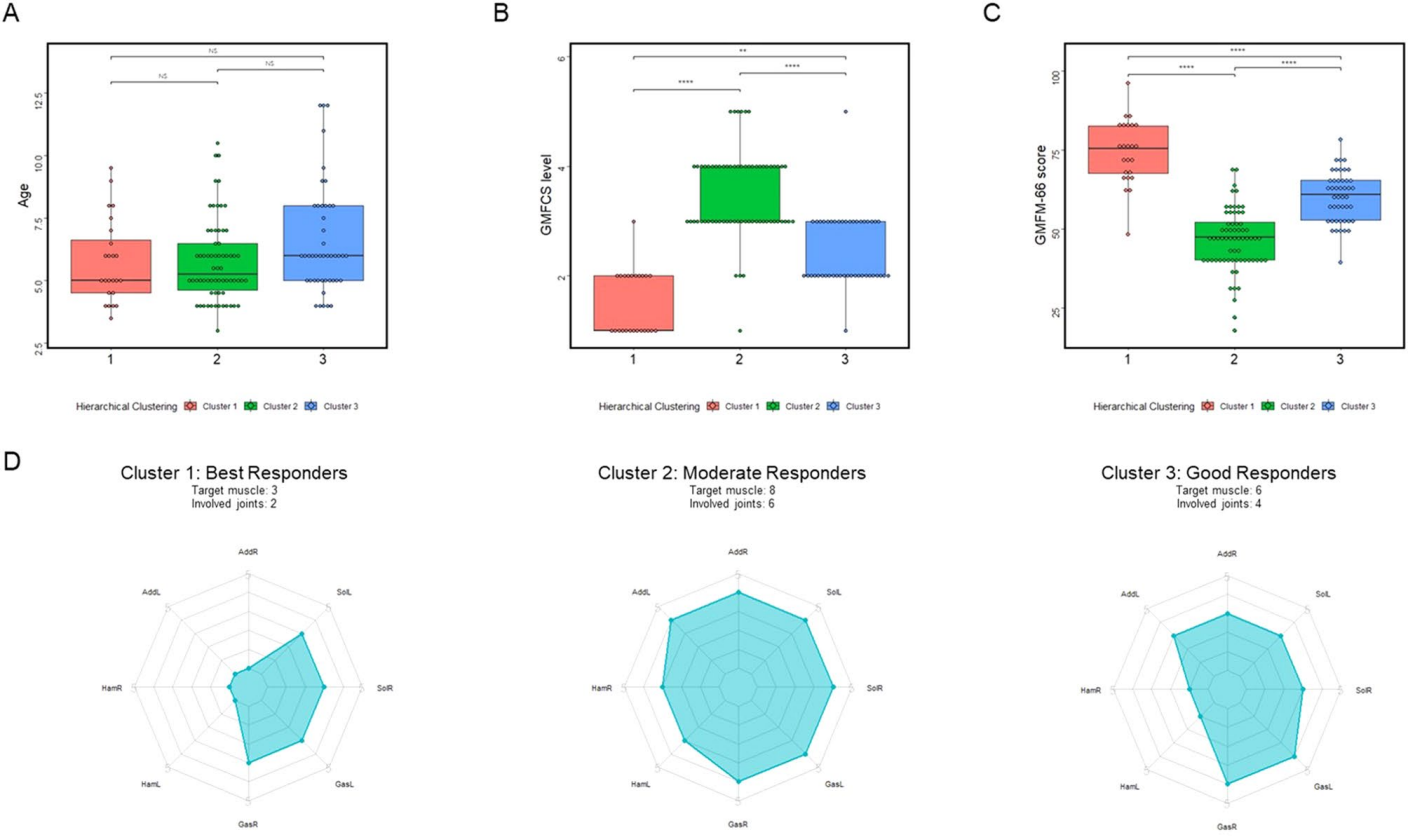
Clinical characteristics of subgroups clustered by hierarchical clustering method. (**A**) Comparison of age at SDR procedure among three clusters. The statistical comparison method used is the Kruskal–Wallis’s test with Dunn's multiple comparison test. The *p* value of the Kruskal–Wallis’s test comparison is 0.11, and the Kruskal–Wallis’s statistic is 4.414. The adjusted *p* value of Dunn's multiple comparison test is shown in the graph using symbols. (**B**) Comparison of GMFCS level at SDR procedure among three clusters. The statistical comparison method used is the Kruskal–Wallis’s test with Dunn's multiple comparison test. The *p* value of the Kruskal–Wallis’s test comparison is less than 0.0001, and the Kruskal–Wallis’s statistic is 75.46. The adjusted *p* value of Dunn's multiple comparison test is shown in the graph using symbols. (**C**) Comparison of GMFM-66 score at SDR procedure among three clusters. The statistical comparison method is ANOVA, and Tukey's test is used for multiple comparisons. The *p* value of the ANOVA comparison is less than 0.0001, and the F value is 85.64. The adjusted *p* value of Tukey's test is represented by symbols in the figure. (**D**) Comparison of MAS score at SDR procedure among three clusters with radar chart. *GMFCS* gross motor function classification system, *GMFM-66* gross motor function measure-66, *AddR* right adductor, *AddL* left adductor, *HamR* right hamstring, *HamL* left hamstring, *GasR* right gastrocnemius, *GasL* left gastrocnemius, *SolR* right soleus, *SolL* left soleus. *NS* no statistical significance. *p < 0.05, **p < 0.01, ***p < 0.001, ****p < 0.0001.

Three subgroups clustered by hierarchical clustering method showed no difference in age, with the mean value of age 5.8 in the first subgroup, 5.8 in the second subgroup and 6.7 in the third subgroup, respectively (Fig. 2). Significant difference existed in the pre-operational GMFM-66 score, with the highest score of 74.3 ± 10.2 in the first subgroup, lowest score of 46.2 ± 9.9 in the second subgroup and 60.0 ± 8.1 in the third subgroup. The median GMFCS level was level 1, level 3, and level 2 in these three subgroups respectively. Patients in the first cluster had the lowest number of target muscles. The median MAS scores of gastrocnemius and soleus were 3 in both sides, and the median MAS scores of bilateral adductors and hamstrings were 0. Children in the second cluster had the highest number of target muscles, the median MAS scores of bilateral hamstrings was 3, and the median MAS scores of adductors, gastrocnemius and soleus were 4 in both sides. The median MAS scores of bilateral adductors and soleus were 3, the median scores of bilateral gastrocnemius were 4, and the median MAS scores of bilateral hamstrings were 1 in the third subgroup.

**Figure 3:**
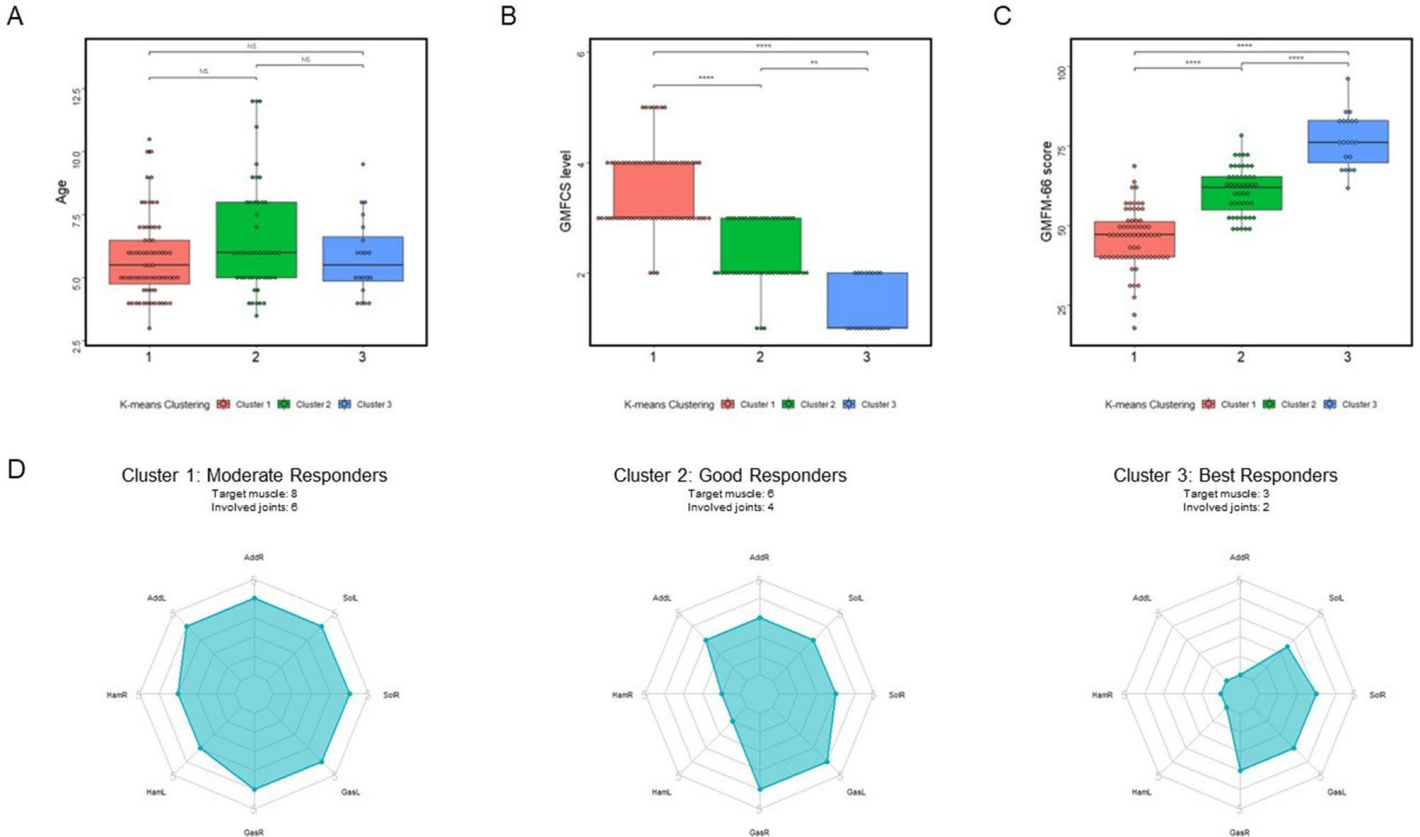
Clinical characteristics of subgroups clustered by K-means clustering method. (**A**) Comparison of age at SDR procedure among three clusters. The statistical comparison method used is the Kruskal–Wallis’s test with Dunn's multiple comparison test. The *p* value of the Kruskal–Wallis test comparison is 0.28, and the Kruskal–Wallis statistic is 2.552. The adjusted *p* value of Dunn's multiple comparison test is shown in the graph using symbols. (**B**) Comparison of GMFCS level at SDR procedure among three clusters. The statistical comparison method used is the Kruskal–Wallis’s test with Dunn's multiple comparison test. The *p* value of the Kruskal–Wallis test comparison is less than 0.0001, and the Kruskal–Wallis statistic is 83.81. The adjusted *p* value of Dunn's multiple comparison test is shown in the graph using symbols. (**C**) Comparison of GMFM-66 score at SDR procedure among three clusters. The statistical comparison method is ANOVA, and Tukey's test is used for multiple comparisons. The *p* value of the ANOVA comparison is less than 0.0001, and the F value is 111.6. The adjusted *p* value of Tukey's test is represented by symbols in the figure. (**D**) Comparison of MAS score at SDR procedure among three clusters with radar chart. *GMFCS* gross motor function classification system, *GMFM-66* gross motor function measure-66, *AddR* right adductor, *AddL* left adductor, *HamR* right hamstring, *HamL* left hamstring, *GasR* right gastrocnemius, *GasL* left gastrocnemius, *SolR* right soleus, *SolL* left soleus. *NS* no statistical significance. *p < 0.05, **p < 0.01, ***p < 0.001, ****p < 0.0001.

Similarly, three subgroups were clustered by K-means clustering method (Fig. 3). No difference was found in age, with an average of 5.8 in the first subgroup, 6.5 in the second subgroup and 5.8 in the third subgroup. The mean pre-operational GMFM-66 score was 45.8, 61.0 and 76.7 in the first, second and third cluster, respectively. The median MAS scores of bilateral hamstrings were 3, and the scores of adductors, gastrocnemius and soleus were 4 in both sides in the first cluster. The median MAS scores of bilateral hamstrings were 1, and the scores of adductors, gastrocnemius and soleus were 3 in both sides in the second cluster. In the third subgroup, the median MAS scores of adductors and hamstrings were 0, with the score elevated in both gastrocnemius (both sides: score 3) and soleus (left: 2.5, right: 3).

### Change of motor function in different subgroups

**Figure 4:**
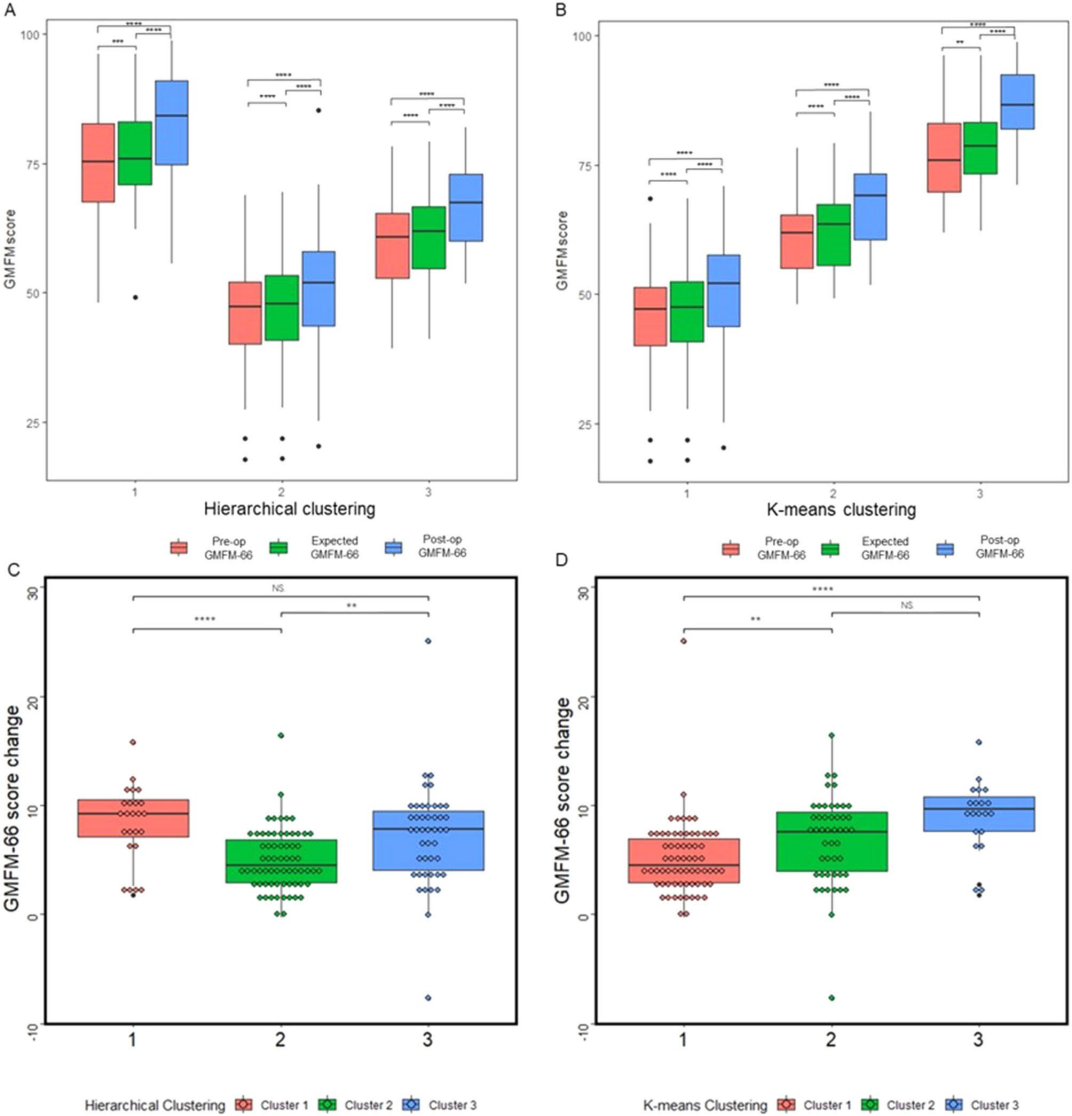
GMFM-66 score change after SDR in three subgroups clustered by hierarchical and K-means clustering algorithms. (**A**) Pre-operational GMFM-66 score, expected GMFM-66 score at follow up and post-operational GMFM-66 score at follow up in subgroups clustered by hierarchical clustering algorithm. Statistical comparison was performed using matched ANOVA and multiple comparison was conducted using Tukey's test. The *p* values and F-values for ANOVA comparisons in Cluster1 were less than 0.0001 and 104.7, respectively; in Cluster2 were less than 0.0001 and 141.7, respectively; and in Cluster3 were less than 0.0001 and 92.04, respectively. The adjusted *p* values for Tukey's test in the three group comparisons were represented by symbols in the figure. (**B**) Pre-operational GMFM-66 score, expected GMFM-66 score at follow up and post-operational GMFM-66 score at follow up in subgroups clustered by K-means clustering algorithm. Statistical comparison was performed using matched ANOVA with Tukey's test for multiple comparison. In Cluster1, the *p* value for ANOVA comparison was less than 0.0001 with an F-value of 109.6; in Cluster2, the *p* value for ANOVA comparison was less than 0.0001 with an F-value of 116.5; in Cluster3, the *p* value for ANOVA comparison was less than 0.0001 with an F-value of 114.7. The adjusted *p* values for Tukey's test were represented by symbols in the figure for the three group comparisons. (**C**) Comparison of GMFM-66 score change in subgroups clustered by hierarchical clustering algorithm. The statistical comparison method used is the Kruskal–Wallis’s test with Dunn's multiple comparison test. The *p* value of the Kruskal–Wallis test comparison is less than 0.0001, and the Kruskal–Wallis statistic is 22.54. The adjusted *p* value of Dunn's multiple comparison test is shown in the graph using symbols. (**D**) Comparison of GMFM-66 score change in subgroups clustered by K-means clustering algorithm. The statistical comparison method used is the Kruskal–Wallis’s test with Dunn's multiple comparison test. The *p* value of the Kruskal–Wallis test comparison is 0.0001, and the Kruskal–Wallis statistic is 25.35. The adjusted *p* value of Dunn's multiple comparison test is shown in the graph using symbols. *GMFM-66* gross motor function measure-66. *NS* no statistical significance. *p < 0.05, **p < 0.01, ***p < 0.001, ****p < 0.0001.

The changes of GMFM-66 score in three subgroups clustered by both hierarchical and K-means clustering methods were compared at the last follow-up (Fig. 4). The results did not show any differences in post-operative follow-up duration between the three groups. The follow-up durations for the three clusters grouped by hierarchical clustering method were 528 ± 158, 573 ± 181, and 581 ± 170 days, respectively, whereas the follow-up durations for the three groups were 565 ± 171, 586 ± 178, and 534 ± 156 days, respectively, under the K-means clustering method. Among subgroups clustered by the hierarchical clustering method, the GMFM-66 scores at last follow-up (82.6 ± 10.8 in cluster 1, 51.2 ± 11.4 in cluster 2 and 67.1 ± 8.1 in cluster 3, respectively) were significantly higher than the pre-operational status (74.3 ± 10.2 in cluster 1, 46.2 ± 9.9 in cluster 2 and 60.0 ± 8.1 in cluster 3, respectively) and the scores expected to be achieved at the follow-up time (75.7 ± 9.9 in cluster 1, 47.5 ± 10.1 in cluster 2 and 61.5 ± 8.4 in cluster 3, respectively) in all three clusters. The promotion is much higher in the first subgroup with median value of 9.2 and third subgroup with median value of 7.8 when compared with the elevation in the second subgroup with median value of 4.5. In all three subgroups clustered by K-means clustering method, the GMFM-66 score at last follow-up (51.0 ± 10.7 in cluster 1, 67.8 ± 8.4 in cluster 2 and 85.8 ± 8.1 in cluster 3, respectively) was significantly higher than the pre-operational status (45.8 ± 9.4 in cluster 1, 61.0 ± 7.6 in cluster 2 and 76.7 ± 8.5 in cluster 3, respectively) and the expected score in all three subgroups (47.1 ± 9.7 in cluster 1, 62.4 ± 7.9 in cluster 2 and 78.3 ± 7.9 in cluster 3, respectively). The elevation in GMFM-66 score is the lowest in the first cluster with median value of 4.5 and highest in the third cluster with median value of 9.6.

### Consistency of two clustering methods

Three subgroups clustered by two methods were named as “best responders”, “good responders” and “moderate responders” in an order from highest to lowest basing on the post-operative GMFM-66 score promotion. Consistency of these two algorithms in clustering the dataset was evaluated by kappa test (Table 2). The kappa value of these two sorting methods was 0.915 with a *p*-value < 0.0001, indicating high consistency of the methods.

**Table 2:** Comparison of the consistency of hierarchical clustering and K-means clustering for patient grouping, kappa value equals 0.915, *p* value < 0.0001.

Hierarchical clustering	K-means clustering		
	Best responders	Good responders	Moderate responders
Best responders	20	4	0
Good responders	0	43	2
Moderate responders	0	1	65

## Discussion

Cerebral palsy is a group of disorders that affects the ability to move, maintain posture and balance^23,24^. Patients diagnosed with SCP are commonly resulted from perinatal hypoxia^25^. Brain magnetic resonance imaging tests of SCP patients mainly exhibit peri-ventricular leukomalacia, which was observed in most of the cases included in the present study. SCP clinical manifestations vary in different patients. SCP patients present with various topographical types (hemiplegic, diplegic and quadriplegic types) and exhibit different degrees of mobility restriction caused by elevated muscle tone. SDR is an effective neurosurgical procedure for alleviation of spasticity in lower limbs and is used for treatment of SCP patients^2,7^. In this current study, it is observed that the spasticity of muscles in lower extremities reduced significantly after the surgery, confirming the surgical effect in relieving muscle tone.

Pre-operational assessment of patients is essential, and it is applied by neurosurgeons to determine patients eligible for SDR surgery^4^. The findings of the current study showed that the patient’s motor function significantly improved after SDR procedure with supplementation of the regular rehabilitation, which is consistent with results from previous studies. Differences were observed in post-operative improvement of patients during the follow-up, though all patients met the indications for the surgical procedure. The purpose of this study is to estimate the short-term motor function change by pre-operational physical assessment and other parameters. This might help to select best candidates for SDR. Machine learning is a recently emerging data analysis approach widely used for analysis of data collected in various fields, which was used in the current study for classification of the patients into different subgroups to identify optimal responders to SDR^26^. Owing to the fact that there’s no standards defining “good” or “bad” surgical outcome, the patients could not be labeled, thus the supervised machine learning could not be used. Patients can only be categorized into various groups based on the similarity of pre-operational clinical characteristics by unsupervised machine learning, and then the surgeons might be able to predict the outcome of the subjects. Postoperative assessment was conducted to evaluate the effectiveness of the clustering method. This method differs from a previous data analysis approach as it compares patients with different outcomes, which is critical in clinical set-ups.

Pre-operational assessment scores of the cases were used as the parameters for the machine learning process in this study. The results showed that unsupervised machine learning through hierarchical and K-means clustering methods effectively and accurately categorized all patients into three subgroups. Principal component analysis dimensionality reduction was conducted to visualize the data and data were compared among the three clusters to validate the accuracy of the classification methods. Principal component analysis is mainly used to downscale data from high-dimensional space to low-dimensional space for utilization in subsequent calculations^22^. The visualization by principal component analysis showed clear cluster boundaries among different clusters, demonstrating the validity of the two clustering methods. After the clustering, the change of GMFM-66 after SDR showed that different surgical outcome existed among three subgroups clustered by both clustering algorithms. As the motor function promotion at post-operational follow up is much higher than the expected natural evolution of motor function, it is confirmed that SDR is helpful to all the patients with SCP who met surgical indications in this study. The different levels of improvement in three subgroups suggested that all the patients could be categorized into three subgroups, best SDR candidates, good SDR candidates and moderate SDR candidates, in an order basing on the level of post-operative motor function promotion. Analysis of the clinical characteristics of the participants showed that the pre-operational GMFM-66 scores of patients defined as best SDR candidates were the highest relative to that of the other two clusters. They also had the lowest number of target muscle groups and relatively mild degree of spasticity, which in detail, the number of target muscle groups less than 4 and the MAS score of target muscles less than 4. On the contrary, patients defined as moderate SDR candidates exhibited the most severe clinical manifestations, namely, the number of target muscle groups at 8 and the MAS score of target muscles mostly greater than 4.

Various machine learning methods use different algorithms and principles. Hierarchical clustering method was used for classification of patients in this study. Classification by hierarchical method is achieved by continuous merging of clusters from the bottom to the top or sorting out clusters from top to bottom^27^. The principle of the algorithm used in hierarchical method is relatively simple. Data are presented as clusters, and the two closest clusters are merged into one cluster until all clusters are combined into a single cluster. Hierarchical clustering results are presented as a dendrogram. In addition, K-means clustering method was used for grouping of subjects in this study. In the K-means clustering approach, a number K is determined by elbow method, and then the computer divides the dataset into K clusters. The algorithm achieves clustering by randomly assigning a number (1 to K) to each data. Subsequently, the cluster centroid for each cluster is computed and each dataset is assigned to the cluster with the closest centroid^21^. These two clustering methods were utilized in classification of patients as they are relatively easy to understand and are widely used in scientific fields. The results indicated the feasibility of the two strategies. However, differences between the two methods were observed in some patients (7/135). Nonetheless, the two methods were effective in sorting the patients enrolled in this study as the consistency of these two clustering methods was high.

Other data clustering methods, such as DBSCAN have been reported^28^. The DBSCAN was also used to classify the dataset in this study. However, the clustering results were not satisfying which were not fully discussed in this paper (Supplementary Fig. 3). Choosing the appropriate method for data analysis is essential for obtaining reliable results.

This study was a single center retrospective study. A relatively small sample size, short follow-up duration and the retrospective nature of the study limit application of the study findings. Moreover, post-operative rehabilitation variables were not included as input variables when classifying the patients due to the challenges in quantification of these variables. Exclusion of post-operative rehabilitation variables in classification may affect the accuracy of the classification results. However, all cases included in this study received standard post-operative rehabilitation therapy; therefore, exclusion of the variables may not have significantly affected the results. What’s more, owing to the nature of unsupervised machine learning, we didn’t split the whole dataset into a training and testing set. Instead, we used the change of GMFM-66 score after SDR to evaluate whether the clustering have clinical significance. Further research should be conducted with a larger population and longer follow-up duration with participants recruited from several centers to verify the validity of the study findings.

## Conclusion

SDR could relieve the spasticity and promote motor function of patients with SCP. Unsupervised machine learning is a feasible and effective method for clustering SCP patients based on pre-operative characteristics. K-means and hierarchical methods effectively and accurately grouped the patients included in this study. Patients clustered by machine learning approach had different pre-operational clinical features and varying levels of improvement in post-SDR motor function, indicating that the clustering approach can be accurately used for prognosis of SCP patients. The results indicate that machine learning is an effective method for determining eligibility of SCP patients before undergoing SDR surgery.

## Supplementary Information

Supplementary Figures.

## Data Availability

The datasets used and analyzed during this study are available from the corresponding author on reasonable request.
