# Supplementary material for: Unsupervised machine learning effectively clusters pediatric spastic cerebral palsy patients for determination of optimal responders to selective dorsal rhizotomy
Source: Sci Rep. 2023 May 19;13:8095. doi: 10.1038/s41598-023-35021-x (PMC10199040; doi:10.1038/s41598-023-35021-x)
Supplement: Supplementary file 1 — Supplementary Figures. [file 41598_2023_35021_MOESM1_ESM.docx]

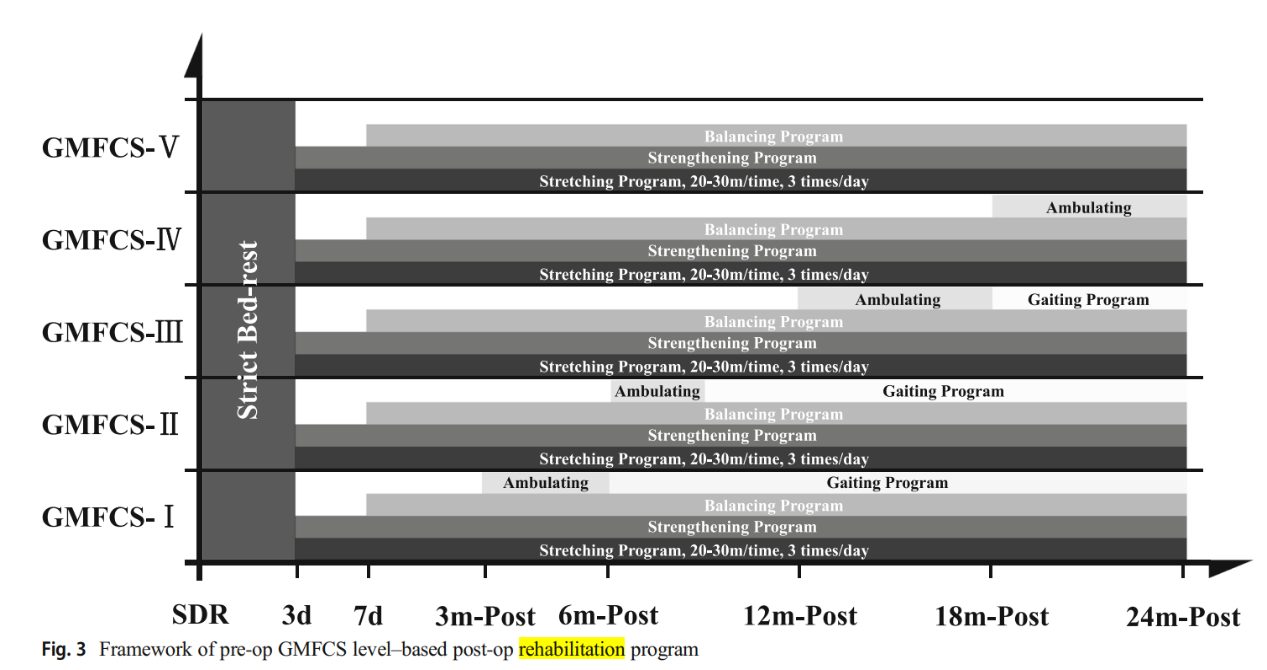


Supplementary Figure 1. Framework of the post-SDR rehabilitation program based on the pre-operational GMFCS level.


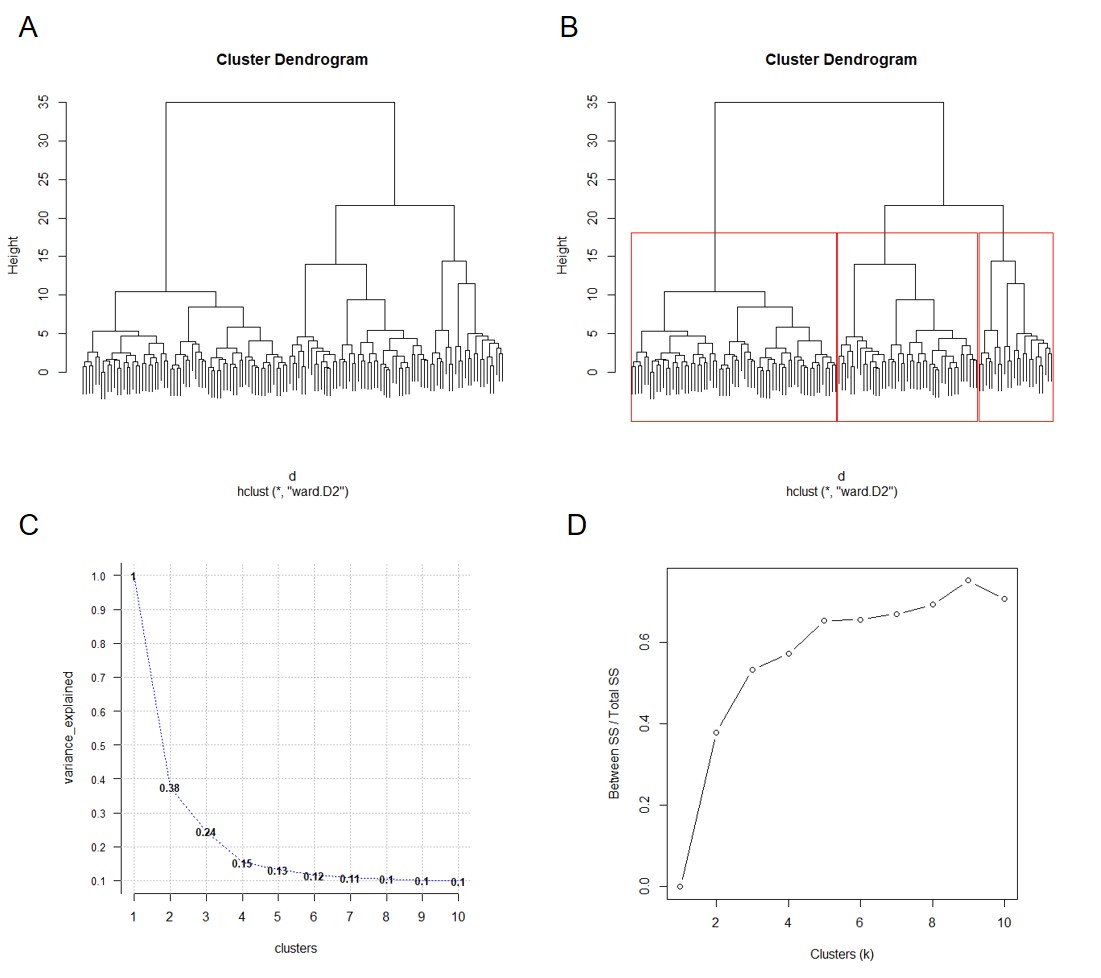


Supplementary Figure 2. Clustering details of hierarchical clustering and K-means clustering.

A. Clustering all included cases by hierarchical clustering method.

B. Based on the visualization of the dendrogram obtained from hierarchical clustering, all cases can be clustered into 3 subgroups.

C & D. Both the elbow plot and the ratio of Between Group Sum of Squares/Total Sum of Squares (Between SS / Total SS) suggest that using K-means clustering to divide all cases into 3 clusters is the best choice.


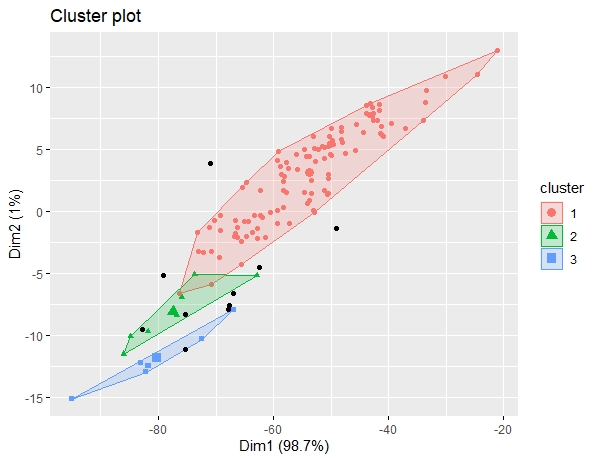


Supplementary Figure 3. Clustering result of DBSCAN (Density-Based Spatial Clustering of Applications with Noise).

Two R packages, “factoextra” and “fpc”, were used to perform DBSCAN clustering on all enrolled cases. The results showed that all patients could be divided into three groups. The black dots represent the noise points that were labeled as such by the DBSCAN algorithm.
